# A Mixed-methods Study on Prosthesis Use Among Older Canadians With Lower-limb Amputations

**DOI:** 10.33137/cpoj.v4i1.36833

**Published:** 2021-08-16

**Authors:** B Pousett, C Budzinski, D Labbé, WC Miller

**Affiliations:** 1 Barber Prosthetics Clinic, Vancouver, British Columbia, Canada.; 2 Holy Family Hospital, Providence Health Care, Vancouver, British Columbia, Canada.; 3 Department of Occupational Science and Therapy, Faculty of Medicine, University of British Columbia, Vancouver, British Columbia, Canada.; 4 Rehabilitation Research Program, Vancouver, British Columbia, Canada.; 5 Department of Disability and Human Development, University of Illinois at Chicago, Chicago, Illinois, USA.

**Keywords:** Prosthesis, Rehabilitation, Lower Limb Amputation, Prosthetist, Older Canadians, Amputation

## Abstract

**BACKGROUND:**

The prevalence of lower-limb amputations (LLA) in older adults is increasing. Due to the substantial resources required for rehabilitation, there is growing interest in exploring prosthesis use as well as the factors that impact use for individuals with LLA.

**OBJECTIVES:**

To examine how older adults, those over 50 years old, with a new LLA use their prostheses following rehabilitation and to identify factors that increase or decrease prosthesis use after discharge from a rehabilitation hospital.

**METHODS:**

The StepWatch Activity Monitor, the Prosthetic Profile of the Amputee Questionnaire, and a semi-structured interview were used to measure prosthesis use and factors affecting use at 12 weeks post-discharge from a rehabilitation hospital. Descriptive statistics were calculated for the quantitative data and the qualitative interviews were analyzed using the International Classification of Functioning and Disability.

**RESULTS:**

Two user profiles emerged from the 11 participants’ use patterns. The Regular Users integrated their prosthesis into their lives, using it for various types of activities, while the Strategic Users wore their prosthesis to perform specific activities of daily and instrumental activities of daily living tasks. Body functions (e.g., pain), personal (e.g., feeling of independence), and environmental factors (e.g., home adaptations or social support) impacted prosthesis use.

**CONCLUSIONS:**

The emergence of profiles suggests variability in how older adults with LLA use their prosthesis after rehabilitation. However, the factors affecting prosthesis use were similar between the profiles. Therefore, while it is important for rehabilitation teams to consider patients’ individual needs when setting goals for prosthetic training, they must also consider common factors affecting prosthesis use.

## INTRODUCTION

In 2011, 7708 lower limb amputations (LLA) were performed in Canada, largely on individuals between the ages of 50-74 (54.9%) due to complications from diabetes (65%).^1^ More than half of these amputations were major amputations (from ankle disarticulations to pelvis amputations) with transtibial amputations being the most common (30.9%).^1^ However, only approximately 20% of individuals with major amputations received inpatient rehabilitation, which provides multidisciplinary care to help integrate the prosthesis into their daily life to improve functional mobility and social participation.^2^ Moreover, prosthetic rehabilitation requires significant health care and economic investments, especially for older adults^3^ thus increasing interest in exploring the breadth of prosthetic use after discharge both in terms of quantity (e.g. wear time) and quality (e.g. type of use, experience).^2,4^

How prosthetic use is defined varies between different studies. Its definition ranged from wear time, functional activity, participation in community or employment, and the number of steps taken.^5-11^ Moreover, the way it is measured varied between studies. Quantitative studies have used activity monitors,^5,6^ functional performance measures,^8,10^ self-reported wear time,^7,8^ and validated questionnaires^7-10^ to measure prosthesis use, while qualitative studies focused more on the type of activities done with the prosthesis, quality of life, and the experience of the prosthesis users.^4,11-14^ The quantitative studies measured prothesis use at different times ranging from discharge to 15 months after rehabilitation and their results varied both in terms of the quantity and quality of prosthesis use.^7-14^ For instance, some studies found a declined of use^6,10^ restricted functioning in mobility,^10^ leisure and social role such as employment,^8,9^ and limited autonomy outdoors while others^7-8^ reported increase prosthesis use overtime and higher functioning in activities of daily living. A qualitative meta-synthesis^13^ of the literature reported that most studies focused on adaptation to the amputation and the prosthesis, the role of social relationships, and the impact of the prosthesis on identity. However, how individuals use their prosthesis in specific life contexts, and the meaning of living with a prosthesis has not been fully been examined.^14^ Moreover, only a few studies^10,11^ combined quantitative measure of use with the qualitative study of the experience and meaning of living with a prosthesis, which likely provides a more nuanced understanding of prosthesis use.

Age has been identified as an important factor affecting prosthesis use.^7,15^ Research has suggested that prosthesis use is influenced by a variety of factors associated with aging. These factors include multiple comorbidities, cognitive impairment, compromised vasculature to the amputated side, significant deconditioning, and fear of falling.^3,11,16^ For instance, Webster et al.^7^ identified that adults older than 55 years old with LLA experienced greater functional restrictions and increased psychological distress than younger individuals. However, many studies only measured self-reported wear time, which did not reflect accurately the activity level.^17^ In addition, these studies did not always collect information about the lived experience of older users, or in what context the prosthesis was used which would help to understand how to support their prosthesis use, that might be different than for younger users. A systematic review concluded that many older prosthesis users demonstrated improved mobility in their home environment, but that more research was needed regarding prosthesis use of older adults in the community.^3^

To better understand the context prosthesis use, it is important to document the factors that impact it. The individual and environmental factors affecting prosthesis use have been explored in literature, albeit mostly with qualitative studies. Findings generally suggest that those with increased independence and confidence tended to use their prosthesis more.^10,12,15,18-20^ Additionally, factors such as support from family and friends, a multidisciplinary healthcare team approach, a strong patient/prosthetist relationship can all positively influence prosthesis use.^7,10,12,20^ Conversely, the presence of pain, multiple comorbidities, impaired balance, decreased cognitive function, emotional challenges, and low daily requirements for standing/ambulating could reduce prosthesis use.^9,12,19^ Moreover, one recent study conducted in Australia20 suggested that terrain, climate, and transport systems could contain barriers to community walking for prosthesis users of all ages. However, such factors have not yet been explored in a Canadian context, which has different physical environments and rehabilitation systems. Moreover, studies included individuals with amputations of all ages and did not necessarily address factors that were unique to older adults with amputations.^15,21^

### Purpose of Study

Building on previous research,^10,11^ this study used a mixed method approach to examine both prosthesis use quantitatively and the lived experience of older adults, defined to be over the age of 50. It also sought to identify personal and environmental factors that increase or decrease prosthetic use in the community after discharge from a rehabilitation program. This mixed method approach provides a deeper and more nuanced understanding^10^ of prothesis use by older adults with LLA.

## METHODOLOGY

### Study Design

This study employed a mixed methods approach to examine prosthesis use, including the frequency of steps taken by participants, how they use their prosthesis and their experience of using a prosthesis. The research ethics boards of the University of British Columbia Ethics Review Committee approved the study protocol.

### Participants

Participants were older adults with LLA who were completing inpatient prosthetic rehabilitation at a Canadian rehabilitation hospital. Participants had to: 1) be over 50 years of age when they acquired their amputation, 2) have a unilateral transtibial or transfemoral amputation, 3) be fitted with their first prosthesis, 4) be participating in the individualized inpatient prosthetic rehabilitation program, and 5) speak English or be able to participate using an interpreter. The age inclusion criteria was chosen because more than half of the people with LLAs in Canada are between ages 50–74 and they often present comorbidities associated with aging.^1^ All eligible participants had recently acquired their amputation and had been assessed by an interdisciplinary team, consisting of a physiatrist, physiotherapist, occupational therapist, social worker and prosthetist, and deemed to be a prosthetic candidate.

Participants who were not deemed to be a prosthetic candidate were not included in this study. Eligible inpatients were invited to participate through a brochure and those interested met with a physical therapist (second author) for more information. Informed written consent was provided by all participants.

### Data Collection

Data were collected from the week prior to discharge (T0) to 12 weeks after discharge (T2). Data collection included demographic and medical information, the StepWatch Activity Monitor (Modus Health Washington, USA), The Prosthetic Profile of the Amputee questionnaire,^22^ and individual semi-structured interviews.

The StepWatch Activity Monitor (SAM) (Modus Health Washington, USA) was used to objectively measure prosthesis use midway through the study (T1) and at 12 weeks post discharge from inpatient rehabilitation (T2), as it was anticipated that users had then acclimated to living in their home environment. These data included the number of steps per day, minutes of active prosthetic use, and the number of days steps occurred on for each participant. The Stepwatch was placed on the participant's prosthesis (around the distal pylon) at T0 and collected data until it was removed at T2. Data were downloaded at six weeks (T1) as the StepWatch can only store 50 days of data. The StepWatch was chosen as it is an accurate tool for measuring step count and activity over an extended period of time for people with a variety of conditions.^23-25^ The reliability and validity of the StepWatch have been established for people with amputations and it has been found to be over 99% accurate.^26,27^

The Prosthetic Profile of the Amputee questionnaire (PPA)^22,28^ is comprised of 44 closed-ended questions, divided into six sections. The PPA was administered at T0 and T2. Information from the PPA was used to complete the medical records on physical conditions and the prosthesis (Q1-9), home environment (Q24-30), and other demographics such as level of education and occupation (Q40-44). The PPA was also used to measure prosthesis use, which includes activities done with the prosthesis, the wear time, and the reasons affecting prosthesis use (Q10-17). Finally, the PPA questions on the use of the prosthesis for leisure and social activities (Q34 to 39) at T2 was used to complement the information gathered during the interviews. Some questions of the PPA did not apply to the participants in this study (i.e., Q22-23 are only completed if the individual does not use a prosthesis) while others were redundant (i.e., both the StepWatch and the PPA (Q12) record how many days a week the prosthesis is worn) and were not included in the analysis. The PPA has an excellent test-retest reliability (between 0.80 and 0.92).^29^ It was administered in person or over the phone. The PPA was pilot tested with two older adults with LLA to verify if using the self-completed online version was an option. They had a hard time completing it by themselves and left many questions blank, the team thus decide to use the paper version in person. Pilot test data were not included in the analysis.

The semi-structured interview was conducted at T2 to learn more about the participants’ experience with prosthesis use. The interview was developed by the research team and it was piloted with two older adults with LLA. The pilot data were not included in the analysis. The interview was conducted by the third author who has extensive qualitative expertise. The interview included open-ended questions to explore: 1) the activities the participant was using their prosthesis for (e.g., “Can you share with me what your typical day looks like? Can you please share with me what it is like for you to wear a prosthesis?”); 2) the personal and environmental factors facilitating or hindering these activities (e.g., “Are there are certain things that encourage you to wear your prosthesis more?”); and 3) participants’ experience with the rehabilitation professionals (“Since going home have you had any outpatient physiotherapy? If yes, how were these visits helpful?”). The interviews were conducted at T2 with each participant either in person (n=8) or over the phone (n=3). They were audio recorded and lasted between 20 to 40 minutes.

Demographic and medical information was extracted from hospital medical records, including age, sex, marital status, comorbidities, amputation level, length of stay, residence type, and the Montreal Cognitive Assessment (MoCA) scores.

### Data Analyses

From the StepWatch data, the number of steps per day was calculated for the prosthetic side only, therefore the total number of steps per day is twice the StepWatch collected value. The data from the 5^th^ week (T1) as well as the seven days before 12 weeks (T2) are presented in this paper. Data from an adjacent week was chosen for two participants (#5 and #9) who were unable to wear their prosthesis for the last week of the study due to medical complications or issues with the prosthetic fit. The StepWatch also provided information on the number of days on which steps occurred, and wear patterns, particularly what time the participant first used the prosthesis, such as early morning or mid-day, and how activity was distributed throughout the day.

For the questions from the PPA on the physical condition, prosthesis, home environment, demographics, and leisure activities, descriptive scores were computed based on the guidelines for this tool to provide descriptive information.^22^ In terms of prosthesis use, scores for question 11, which assesses independence during a variety of tasks, were calculated for both time points, where higher scores (to a maximum of 42) indicate increased independence. The scores for questions 14 & 16 were only calculated at T2 because these questions were not applicable to inpatient rehabilitation (e.g., What percentage of the time do you use the prosthesis [inside] or [outside]?).

The interviews were transcribed verbatim. A content analysis approach^30^ was used to identify, analyze, and interpret patterns in the participants’ responses. The first five interviews were reviewed by the entire research team and an initial coding grid was developed. The coding grid was created following the International Classification of Functioning, Disability and Health construct (ICF)^31^ to provide codes from the domain of activities and participation to qualify the experience of prosthesis use, and from the domains of Body Functions and Environmental Factors to identify components impacting use. The coding grid also included codes for Personal Factors codes that were mentioned by participants but are not categorized by ICF. Two team members then independently coded the complete interview data set with this coding grid, added codes if needed, and discussed all coding to reach a consensus. The final coding grid was applied to all interviews.

Different trustworthiness strategies were used such as reflective commentary, and triangulation.^32^ The primary author took interview notes to reflect on the interview's context and her initial impressions and personal bias. No member checking was done. Data triangulation was supported using different sources of data (interviews, questionnaires, step watch). The triangulation of different researchers' perspectives during the content analysis also helped ensure credibility.

### Integration of Data and Development of Profiles

Through a series of collaborative discussions between the research team, the entire data set was analyzed and integrated to develop two profiles reflecting prosthesis using styles of older adults with LLA in their community. Those profiles provide an archetypical case studies of prosthesis use that illustrate commonality found among the participants, as used in a previous study on power wheelchair use.^33^ To create each profile, the team grouped the participants based on information from a combination of all the data collected: the number of days worn, steps per day, and observable patterns of use were used; satisfaction and adaptation to the prosthesis and type of usage as measured by the PPA, and based on the narratives by the participants of their usage and experiences with the prosthesis as shared during the interviews.

## RESULTS

### Participants

Between December 2015 and August 2017, 59 older adults with LLA were admitted to the rehabilitation program - 13 consented to participate and 11 completed the study. One participant was discharged unexpectedly and the other withdrew for medical reasons. We achieved theoretical saturation with this number of participants, which suggests we obtained a sufficient number of representative participants to answer the research question.^34^ The participants’ demographic and clinical information are described in **Table 1**. The sample had an equal distribution in terms of sex (6 men,5 women), and the average age was 67.8 years old. This is very similar to the typical population of patients seen at this facility.

**Table 1:** Clinical and sociodemographic characteristics of the participants.

# of participants	Age (yrs)	Sex	Marital Status	Education (yrs)	Occupation	Level of Amputation	Cause of Amputation	Total LOS in rehab hospital (days)	Prosthetic Training (days)	MoCA (on 30)
1	73	Female	Married	>12	Working	Right TTA	Raynauds	167	34	26
2	76	Male	Single	>12	Retired	Left TTA	PVD/Diabetes	100	57	28
4	87	Male	Married	12	Retired	Left TTA	PVD	117	56	16+1
5	69	Male	Married	>12	Retired	Left TTA	Foot deformity CRPS	71	30	24
6	66	Female	Married	>12	Disability	Right TTA	Diabetes	137	37	NT
7	66	Female	Common-law	12	Retired	Left TFA	Infected knee replacement	183	46	25+1
9	52	Female	Single	<12	Retired	Right TTA	Foot deformity Radiculopathy	106	84	24+1
10	73	Female	Married	>12	Retired	Left TTA	PVD	89	56	NT
11	66	Male	Married	12	>12	Working	PVD/Diabetes	120	45	NT
12	60	Male	Married	>12	Retired	Left TTA	PVD/Diabetes	59	29	NT
13	58	Male	Married	>12	Retired	Right TTA	Diabetes/Charcot foot	66	29	26/30

LOS = length of stay; TTA = transtibial amputation; TFA = transfemoral amputation; NT= not tested; PVD = peripheral vascular disease, CRPS = complex regional pain syndrome; +1 on the MoCA score = 12 years of education

From the PPA (Q1-9; Q24-30; 40-44), all participants had at least four co-morbidities with the most common being peripheral vascular disease (PVD), diabetes, hypertension, and low back dysfunction. The main problems with their non-amputated leg were swelling, poor circulation, and cramps. The main problems with the residual limb was phantom pain and a few participants (4 out of 11) also experienced occasional pain. The majority of participants (9 out of 11) were quite well or completely satisfied with the comfort, appearance, and weight of their prosthesis as well as the appearance of their gait while using it. However, they experienced some issues with their prosthesis—mainly skin irritation and excessive stump perspiration. All the participants lived in a house or apartment, with half of them having no stairs in or outside their home.

### Are older adults with LLA using their prosthesis?

**Figure 1:**
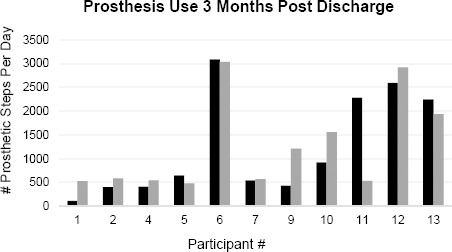
Daily average number of steps on the prosthesis at T1 (black) and T2 (grey).

**Figure 2:**
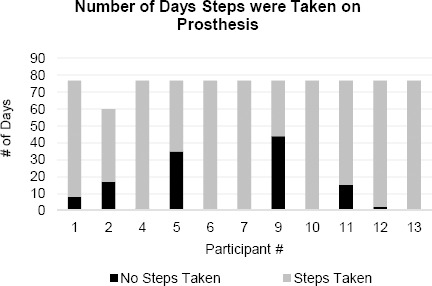
Number of days that participants took steps with their prosthesis. Black represents days with no steps taken and grey represents days with steps taken.

All individuals in this study used their prosthesis regularly, though wear patterns differed between participants. The daily average number of steps taken on the prosthesis at T2 ranged from 481 to 3031 (**Figure 1**). Some participants walked with their prosthesis every day while others had several days where no steps were taken (**Figure 2**). It should be noted that one participant (#11) experienced a foot injury towards the end of the study, resulting in a day in which there was a large decrease in steps. For one participant (participant 2), there were several days where the StepWatch was put on the prosthesis upside down and data were not recorded.

**Table 2** presents the use of the prosthesis based on the PPA. First, a mobility score was calculated using the PPA. The mobility score represents an individual’s ability to complete a series of activities while wearing their prosthesis and how much help they required to do so. The PPA provided additional information about how participants used their prosthesis. It revealed that four participants were part time wheelchair (WC) users, mostly inside their homes (i.e., used the WC 50 to 75% of the time). The main reasons for WC use given by the participants were other health conditions such as back or respiratory issues, and problems with the prosthesis.

**Table 2:** Select questions from the Prosthetic Profile of the Amputee questionnaire.

	Q.11 Mobility function score (Max score of 42)			Q. 14 Indoor mobility (T2) (%)		Q. 16 Outdoor mobility (T2) (%)		
No.	In Hospital (T0)	At Home (T2)	In W/C	Walking with prosthesis	Walking without prosthesis	In W/C	Walking with prosthesis	Walking without prosthesis
1	22	24	75%	0%	25%	100%	0%	0%
2	24	32	75%	25%	0%	0%	100%	0%
4	33	31	25%	75%	0%	0%	100%	0%
5	27	42	50%	50%	0%	0%	100%	0%
6	31	33	25%	75%	0%	0%	100%	0%
7	36	40	0%	100%	0%	0%	100%	0%
9	42	41	50%	50%	0%	50%	50%	0%
10	32	41	0%	100%	0%	0%	100%	0%
11	42	42	25%	75%	0%	25%	75%	0%
12	40	42	0%	100%	0%	0%	100%	0%
13	33	39	0%	100%	0%	0%	100%	0%

T0 = 1 week prior to discharge, T2 = 12 weeks post-discharge, W/C = wheelchair

### How are older adults with LLA using their prosthesis?

When considering how participants were using their prosthesis, two distinct user profiles emerged: the Regular Users and the Strategic Users.

### Regular Users

Regular Users (participants 4, 6, 7, 10, 11, 12, and 13) had integrated their prosthesis into their lives. They wore their prostheses from morning to evening, 12 to 16 hours a day, seven days a week. The intensity of activity was consistent throughout the day and they took 400 - 3000 steps a day on their prosthesis. The PPA showed that Regular Users used their wheelchairs less to mobilize inside their homes and used their prosthesis almost exclusively for outdoor mobility. These users also reported higher levels of mobility function and independence.

In the interviews, these participants emphasized the frequent use of their prosthesis through comments such as *“I put it on the first thing in the morning and I take it off the last thing at night”* (#7, a 66 year-old female with a left TFA). The Regulars Users wore their prosthesis for a variety of activities: mobility, self-care, and domestic life. In terms of mobility, they used their prosthesis to walk short and long distances, moving around in and outside their homes, *“I haven’t had a chance to go for a field run with my new prosthetic from yesterday, but I was up to being able to hit about 3, 4 kilometers before that was enough for me for a day”* shared #12, 60 years-old male with a left TTA. Participants also mentioned regularly wearing their prosthesis to conduct self-care activities such as toileting and dressing. In terms of domestic life, they used their prosthesis when preparing meals or cleaning. Regular Users also conducted community, social, and civic life activities such as shopping, going to the restaurant, or the church using their prosthesis. For instance, one 73 years old women with a left TTA (#10) explained *“I will do some sewing, or I will play the piano […] I’ll try to do a little walking in the back garden if the weather is good, or I will do my exercises. And in the afternoon […] If the weather is not too bad, we’ll go out for a walk… usually, we’re outside for maybe an hour and a half and then we stop sometimes to the grocery store on the way back”.* The Regular Users were using their prosthesis both for sports (e.g. golf, curling, yoga) and leisure activities (e.g., arts and crafts or gardening) as seen in their answers to the section on leisure activities in the PPA and their narratives from the interviews. The Regular Users began to see their prosthesis as part of themselves, as one participant said (12, man, 60 years old, with a left TTA) *“Me and my legs are the same things.”*

### Strategic Users

Strategic Users (participants 1, 2, 5, and 9) viewed their prosthesis as a tool to complete specific tasks and activities. They used their prosthesis for shorter intervals throughout the day: three to seven days a week. From the PPA, these individuals frequently mobilized with a wheelchair inside their homes (>50% of the time) but outdoor wheelchair use varied. Finally, these users reported lower levels of mobility function and independence.

During the interviews, the Strategic Users explained that they donned their prosthesis later in the day or used it only for specific mobility activities such as short distance walking (often as part of their exercise regime) and walking inside the home. They rarely used their prosthesis to move around outside their homes. The Strategic Users also put on their prosthesis for specific self-care activities such as taking care of their health by attending physiotherapy appointments. They also used it for specific domestic life activities such as doing the dishes. They did not use their prosthesis to participate in their social or community life. For instance, one participant (#1, female 73 years old with a right TTA) explained: *“Today I put it on at 10. It depends what I’m doing, if I’m going out. I don’t usually wear to work[…] I take it off for a while and then put it on back on because it gets a bit sweaty at times […] in the afternoon”.* Sometimes stump swelling sometimes forced participants to remove their prosthesis. Some participants indicated they developed a preference for their wheelchair: *“There is nothing so cozy and comforting as a wheelchair”* (#2, a 76 year old male with a left TTA ).

### What factors influenced prosthesis use?

In the interviews, participants identified personal and physical and social environmental factors that limited or increased their prosthesis use. Those factors did not distinguish between the Strategic-and the Regular Users; some factors were described as facilitating use by some participants but as limiting use by others, while other factors were identified as both facilitators and barriers by all participants albeit their type of use.

Regarding personal factors, the feelings of normalcy and increased independence associated with wearing the prosthesis facilitated prosthesis use. One participant (#4, an 87-year-old male with a right TTA) said, *“The prosthesis gives me the freedom to do things”*. Some participants had not yet experienced those feelings but mentioned that regaining their independence and being “normal” again motivated them to wear their prostheses. On the other side, co-morbidities such as diabetes or cardiorespiratory problems were cited as barriers to prosthesis use by all participants.

The factors of the physical environment concerned the assistive technologies and the home environment (product and technology) and climate and weather (natural environment). Regarding assistive technology, the older adults mentioned that using a cane or a walker aided walking with the prosthesis. The participants also felt that the prosthesis facilitated use when it was fitting well, but was a barrier when it was uncomfortable due to poor fit or poor sock management. The location of the home was a facilitator. For instance, one participant reported using more the prosthesis because of the surroundings of his home *“We live on a hillside. So there is just no way that I would be able to use the wheelchair for very much outside”* (#12, man, 60 years old, with a left TTA). Housing modifications made to increase accessibility were also reported as facilitating prosthesis use, especially bathroom adaptations such as handrails and shower benches, or having an elevator inside the residence. Conversely, home feature such as stairs were limiting prosthesis use. Inclement weather such as snow and rain, was perceived as a barrier. Participants said they wished they would have been trained to walk in those conditions with their prosthesis.

In terms of the social environment, receiving support from friends and family was a factor that promoted prosthesis use. One woman (#7, 66 years old with a left TFA) explained during her interview *“Oh, my girlfriends, they always say, ‘oh my gosh, this is the best thing’ and they say ‘you’re really good’.”* Regarding health care services, systems and policies, the participants perceived that the informational and emotional support provided by the healthcare professionals such as the physiotherapists and prosthetists facilitated their prosthesis use. For instance, they perceived their inpatient training as useful—participants appreciated the reinforcement they received about their progress. One older participant *said “They are teaching you how to stand up straight and learn to walk. And then finally you are walking. And you are walking around the gym. And those things are very important […] psychologically important, because they are teaching you that the things can still be as they were. You don’t have to spend the whole rest of your life in a wheelchair.”* (#4, an 87-year-old male with a right TTA). The rehabilitation professional’s attitude and knowledge also motivated participants to use their prosthesis. Participants emphasized that advice regarding sock management and how to adjust their prosthesis fit was particularly helpful.

## DISCUSSION

This study used a mixed-method approach to examine how older adults with LLA use their prosthesis following rehabilitation and to identify factors that increase or decrease prosthetic use after discharge. The creation of the profiles of use, “Regular User” and “Strategic User”, allowed for a holistic portrait of prothesis use by older adults with LLA.

All of the participants in this study used their prosthesis, as measured with step counts, which aided their goals. More than half of the participants took approximately 500 steps per day on their prosthesis which is below the “basal” activity level threshold for older adults.^35^ The Regular Users achieved more than 1,250 prosthetic steps per day, which is considered a “sedentary” activity level, only two achieved the “limited”35 activity level by walking over 2,500 prosthetic steps per day. While step count is known to fluctuate for reasons secondary to personal and environmental factors including seasonal changes, health status, prosthetic fit, and life situations, it gives a snapshot of prosthetic use at a given time point.^5,6,10^ The 5 most active participants took about 3000 total steps per day, which is similar to other studies that found that adults of all ages with LLA or those with diabetes averaged more than 3000 steps per day (3 months after discharge from rehabilitation).^17,36^

Despite their variability in use, all the participants improved their mobility during the time of the research. This is similar to other research that found that prosthesis use in older adults, as measured by reported wear time, increased over time post amputation.^11,16^ Individuals may need more time to experience using the prosthesis in their home environment before truly understanding how they may integrate it into their life, suggesting the importance of considering a long term follow-up (e.g., at least 6 months) in the rehabilitation process.4 Currently, outpatients with LLA in Canada receive four to six weeks of outpatient rehabilitation, a period when the prosthesis still needs adjustments and is not regularly used as our results suggest.^2^ Tools like the PPA^22^ could also be used when the prosthesis user returns for outpatient follow-up to have a more complete and objective outcome measure. Moreover, multiple follow-ups could help older users with LLA consolidate their training by repeating information or discussing problem solving, which could be especially important as older users may experience decreased cognitive functions.^19,21^

The two user profiles, distinguished how participants used their prosthesis in their daily lives. The identification of variance in prosthesis use is similar to previous studies that reported heterogenous use patterns after discharge.^7,16^ For instance, when examining older patients with LLA one year post-discharge from a rehabilitation program, Hershkovitz et al.^16^ found a distribution including three groups: full time use (e.g. most of the days), part time use, and no use. Using mixed methods to identify the Strategic-and Regular user profiles allowed us to explain the identified pattern of use quantitatively by referencing step count and the PPA, as well as qualitatively by exploring lived experiences during interviews. For instance, while the StepWatch revealed that regular users were wearing their prosthesis more and taking more steps throughout the day, the PPA and interviews confirmed that they used their prosthesis for all their activities. Moreover, they perceived their prosthesis as a part of their bodies which suggest a higher level of integration.^13^ On the other hand, the shorter periods of activity found with Strategic Users’ StepWatch data was mostly due to the fact that they used their prosthesis for targeted basic activities of daily living (e.g., going to the bathroom) or exercise (e.g. walking short distances) as explained by the interviews data. The identification of user profiles suggests that rehabilitation professionals could focus on individualized goals of older adults with LLA, as proposed in recent studies.^11,20^ It suggest the need to incorporate various types of training such as ambulating outdoors on uneven ground, ramps, and stairs into rehabilitation programs to improve mobility outside of the home environment – a rewarding experience for many individuals which helps to establish a sense of normalcy.^11^ Working on individualized goals also mean that the rehabilitation professional discusses community participation needs^20^ with prosthesis users. Currently in Canada, few rehabilitation facilities offer return-to-work or recreational therapy that could support these needs.^2^

This study reported several personal and social environment factors that help the older adults using their prosthesis. As the older adults progress through their rehabilitation and increase their mobility, they gained confidence and reported an increased sense of freedom and independence, similar to users of all ages in previous studies.^4,18^ This was especially true for the regular users who integrated their prosthesis into their daily lives and were beginning to see it as part of their body. This feeling of normality and freedom might be a goal for some older users but not necessarily for all. It is thus important for the prosthesis user and other rehabilitation professionals to discuss what normality and independence mean for the users to better support them to reach their “new normal”.^14^ As has been found for younger prosthesis users,^6,10,12,18,20^ support from family and friends positively affect the transition to being a prosthesis user in older age. This suggests that including family and/or caregivers in rehabilitation may be a valuable way to build a support network for the individuals to continue using the prosthesis after being discharged.^12^ Facilitating peer support from those who have already made positive adjustments to amputation and prosthesis use could also be more integrated into the rehabilitation process.^13^ It could show older adults with LLA what is still possible for them to do and to identify goals for themselves that they might not have thought of. Our research also supports similar studies showing the importance of regular follow-ups with prosthetists. Follow-ups helped increase prosthesis comfort and allowed participants to develop a relationship with their prostheses, factors that facilitate use.^10,12,18^

Several personal and physical environmental factors were identified that limited older adult participants from using their prosthesis. Some of these factors such as discomfort in the socket or challenges with outdoor mobility could be mitigated by regular follow-ups with the rehabilitation team. Our study also underlined the impact of inclement weather, such as rain, ice, and snow, adding to previous literature only reporting heat as a climatic barrier.^9,20^ This is especially relevant to individuals who completed their rehabilitation during the summer months with no exposure to wet or slippery environments that are common in Canada and other countries with a similar range of weather patterns. It reaffirms the importance of long-term follow-up rehabilitation to assure that patients do not miss out on community outings due to a lack of seasonal preparation. As argued by vanTwiller et al,^10^ it is inevitable that patients will encounter barriers. However, individual reactions to these perceived barriers could greatly vary. Therefore, it would be helpful to teach older prosthesis users about positive coping strategies to deal with problematic situations, to support their prosthesis use.^13^

### Limitations

The limitations of this study include the sample being a small number of participants from a single rehabilitation setting. This limits the ability of these findings to be applied to other settings and populations. Collecting data from different rehabilitation settings would provide evidence regarding the applicability of these results in other settings. More specifically, this study only includes one participant with a transfemoral amputation and as such the findings are not generalizable to the transfemoral population. In addition, while this study followed participants in depth for the first 12 weeks after discharge from rehabilitation, nothing is known about the participants’ long-term prosthesis use. Collecting additional data (e.g., six months and two years post discharge) could produce rich evidence in the long-term benefits and needs of rehabilitation programs. Future research could also look at individuals from communities where resources and support are limited such as rural communities, or from different cultural backgrounds to see if they have a different pattern of use.

## CONCLUSION

The results of this study showed that older adults with lower limb amputations are using their prosthesis, but also that there is variability in how they are integrated into their daily lives. The older adults in both the regular and strategic users profiles, used their prosthesis to complete basic and instrumental activities of daily living. However, differences existed in how the prosthesis is used during community and social activities. Moreover, factors impacting use were similar across the profiles. Therefore, it is important for rehabilitation teams to work with their patients to recognize potential barriers and provide the tools to problem solve around them, especially once patients can walk and are focusing on higher level activities. Follow-up with the rehabilitation team once the patient is discharged could help ensure the patient’s prosthesis is comfortable and also address new concerns that may arise from prolonged use of the prosthesis at home and in community environments.

## DECLARATION OF CONFLICTING INTERESTS

The Author(s) declare(s) that there is no conflict of interest.

## AUTHOR CONTRIBUTION

- **Brittany Pousett,** contributed to the study design, data management and analyses of the step watch data and writing of the manuscript and study report.
- **Colleen Budzinski,** contributed to the development of the design and protocol, ethics submission, management of the research team, analyses of the data and editing of the manuscript.
- **Delphine Labbé,** contributed to the recruitment, enrollment, data collection, data analyses and writing of the final paper and report. Delphine Labbé is now an assistant professor at the University of Illinois at Chicago.
- **William C Miller,** contributed to the development of the design and protocol, management of the research team, administrative support, analyses of the data and editing of the manuscript.

## SOURCES OF SUPPORT

This work was supported by the Providence Health Research Challenge.

## ETHICAL APPROVAL

This study was approved by the University of British Columbia Ethics Review Committee.
